# Phenylalanine Hydroxylase Misfolding and Pharmacological Chaperones†

**DOI:** 10.2174/1568026611212220008

**Published:** 2012-11

**Authors:** Jarl Underhaug, Oscar Aubi, Aurora Martinez

**Affiliations:** Department of Biomedicine, and K.G. Jebsen Centre for Research on Neuropsychiatric Disorders, University of Bergen, Jonas Lies vei 91, 5009 Bergen, Norway

**Keywords:** Phenylalanine hydroxylase, pharmachaperones, high-throughput screening, pharmacophore modeling, folding aids.

## Abstract

Phenylketonuria (PKU) is a loss-of-function inborn error of metabolism. As many other inherited diseases the main pathologic mechanism in PKU is an enhanced tendency of the mutant phenylalanine hydroxylase (PAH) to misfold and undergo ubiquitin-dependent degradation. Recent alternative approaches with therapeutic potential for PKU aim at correcting the PAH misfolding, and in this respect pharmacological chaperones are the focus of increasing interest. These compounds, which often resemble the natural ligands and show mild competitive inhibition, can rescue the misfolded proteins by stimulating their renaturation *in vivo*. For PKU, a few studies have proven the stabilization of PKU-mutants *in vitro*, in cells, and in mice by pharmacological chaperones, which have been found either by using the tetrahydrobiopterin (BH_4_) cofactor as query structure for shape-focused virtual screening or by high-throughput screening of small compound libraries. Both approaches have revealed a number of compounds, most of which bind at the iron-binding site, competitively with respect to BH_4_. Furthermore, PAH shares a number of ligands, such as BH_4_, amino acid substrates and inhibitors, with the other aromatic amino acid hydroxylases: the neuronal/neuroendocrine enzymes tyrosine hydroxylase (TH) and the tryptophan hydroxylases (TPHs). Recent results indicate that the PAH-targeted pharmacological chaperones should also be tested on TH and the TPHs, and eventually be derivatized to avoid unwanted interactions with these other enzymes. After derivatization and validation in animal models, the PAH-chaperoning compounds represent novel possibilities in the treatment of PKU.

## 1. INTRODUCTION

### Phenylketonuria (PKU)

Phenylketonuria (PKU; OMIM 261600) is an autosomal recessive inborn error of amino acid metabolism, and it is characterized by intolerance to the dietary intake of the essential amino acid phenylalanine (Phe) and consequent elevated levels of Phe in blood and tissues (hyperphenylalaninemia; HPA). PKU was first described in 1934 by Dr. Asbjørn Følling who studied two mentally retarded siblings who excreted phenylketonuric acid in their urine [1]. Følling suggested that they were affected by an inherited defect in the metabolism of Phe. PKU is the most common of the inborn errors of amino acid metabolism, and its incidence varies widely around the world, with approx. 1:10,000 live births in Europe [2]. If left untreated, PKU is accompanied by progressive mental retardation, brain damage, epilepsy, and neurological and behavioral problems caused by a neurotoxic effect of HPA (for recent reviews see [2-6]). Since the late 1960s newborn screening programs using the Guthrie test [7] have ensured early diagnosis of PKU and prompt initiation of a treatment consisting of strict protein-free diet with supplementation of amino acids other than Phe. In most cases, PKU is caused by a deficiency in the first enzymatic step in Phe oxidation due to mutations in the phenylalanine hydroxylase (*PAH*) gene. Information on mutations is provided at the phenylalanine hydroxylase locus knowledgebase (PAHdb) [8] (http://www.pahdb.mcgill.ca), and at the BIOPKU database, which also includes information on allele combinations (http://www.biopku.org). In addition to classical PKU, characterized by plasma Phe levels >1,200 µM in untreated patients, mutations in *PAH* are also associated to mild PKU, also called “atypical” or “variant” PKU, with plasma Phe in the 360–1,200 µM range and moderate Phe tolerance in the diet, and mild HPA (or “non-PKU” HPA), with plasma Phe levels 120–600 µM and high tolerance to Phe in diet [2]. At present, the dietary Phe restriction is adjusted to each patient to maintain their plasma Phe within safe levels, i.e. <360 µM (<240 μM for pregnant PKU women). However, it is difficult for the patients to strictly adhere to the Phe-free diet, which in addition often leads to malnutrition and psychosocial complications [9]. Consequently, alternative treatments have begun to emerge, the most recognized being the PAH cofactor therapy, i.e. treatment with KUVAN^®^ (sapropterin dihydrochloride; tetrahydrobiopterin (BH_4_)) (see below).

### Phenylalanine Hydroxylase (PAH) and Tetrahydrobiopterin (BH_4_)

Phenylalanine hydroxylase (PAH; phenylalanine 4-monooxygenase; EC 1.14.16.1) catalyzes the para-hydroxylation of L-Phe to L-Tyr. This is the first step in the catabolic degradation of L-Phe, and about 75% of the L-Phe from the diet is degraded this way at physiological conditions [10]. The PAH reaction requires (6*R*)-L-*erythro*-5,6,7,8-tetrahydrobiopterin (BH_4_) as natural cofactor Fig. (**1**) and O_2_ as additional substrate. The cofactor BH_4_ is in fact a co-substrate that accepts one of the oxygen atoms from O_2_ and gets hydroxylated in the reaction, generating pterin-4a-carbinolamine (4-OH-BH_4_). After each PAH catalytic cycle, BH_4_ has to be reduced to its functional tetrahydro form by the coupled action of pterin carbinolamine dehydratase (PCD), which catalyzes the dehydration of 4-OH-BH_4_ to quinonoid dihydrobiopterin (q-BH_2_), and of dihydropteridine reductase (DHPR), which catalyzes the conversion of q-BH_2_ back to BH_4_ Fig. (**1**). q-BH_2 _may also rearrange non-enzymatically to 7,8-dihydrobiopterin (BH_2_), which is no longer a substrate of DHPR, but requires the alternative salvage pathway, involving the methotrexate-sensitive enzyme dihydrofolate reductase (DHFR), to be reduced back to BH_4_ [11]. The *phenylalanine hydroxylase system* is generally considered to include the two BH_4_ regenerating enzymes PCD and DHPR in addition to PAH. Moreover, enzymes involved in the *de novo* synthesis of BH_4_ from guanosine triphosphate (GTP) are GTP cyclohydrolase I (GTPCH), 6-pyruvoyltetrahydropterin synthase (PTPS), and sepiapterin reductase (SR) [11]. There are genetic disorders associated to all these enzymatic steps, which cause different degrees of HPA and/or impaired neurotransmitter synthesis (for clinical and molecular data of patients with BH_4_ deficiencies see the BIODEF and BIOMDB databases; http://www.biopku.org). Therapy includes BH_4_ supplementation, usually accompanied by L-DOPA and 5-hydroxytryptophan [11].

PAH belongs to the family of the aromatic amino acid hydroxylases, which includes PAH, tyrosine hydroxylase (TH), and the tryptophan hydroxylases (TPHs) (for reviews see [12-15]). Mammals contain one *PAH* and one *TH* gene, but two *TPH* genes, i.e. *TPH1 *which shows higher expression in peripheral tissues, and *TPH2*, which seems to show a brain and neuron-specific expression [16, 17]. The mammalian aromatic amino acid hydroxylases are tetrameric and each subunit includes three domains: i) an N-terminal regulatory domain, which locates phosphorylatable Ser residues (Ser16 in human PAH), ii) a catalytic domain, that shows a high sequence similarity among the hydroxylases and which holds a catalytic non-heme iron atom through a 2-His-1-carboxylate facial triad motif [18], and iii) a C-terminal oligomerization domain consisting of dimerization and tetramerization motifs [14, 19]. 

The 3D structure of truncated forms of tetrameric rat TH [20] and dimeric human PAH [21] were determined in 1997, and following these breakthroughs there appeared several structures of diverse forms of PAH, also including the regulatory domain [22], and the tetramerization motif [19]. Though the 3D structure of full-length tetrameric PAH has not yet been determined, it is possible to prepare composite molecular models of this form using the structures available so far Fig. (**2A**). The residues coordinating the iron catalytic iron in human PAH are His285, His290, and Glu330, and the location of the binding sites for the substrate and the cofactor or cofactor analogues has also been determined by NMR and molecular modeling [23, 24] and from X-ray crystallography [25, 26] Fig. (**2B**). Moreover, the residues determining the binding selectivity and affinity for the amino acid substrate and the cofactor in PAH, comparative to TH and the TPHs, have been rationally discussed based on comparative analyses of the available crystal structures [13-15]. Furthermore, the advances in the structural characterization of PAH have provided a rational frame to interpret the structure-function relationships and the genotype-phenotype relationships in PKU-associated mutations [27, 28]. 

The catalytic mechanism of the aromatic amino acid hydroxylases has been studied by several groups both experimentally and computationally, notably by density functional theory (DFT) (for reviews see [13, 29]). This mechanism appears to occur by O_2_ binding and activation via a Fe–O–O–BH_4_ bridge, followed by heterolytic cleavage of the O–O bond to form the Fe(IV)=O hydroxylation intermediate, whose existence was proven experimentally [30], and subsequent hydroxylation of the amino acid substrate. 

With respect to regulation of the enzyme activity, tetrameric mammalian PAH is activated several fold by preincubation with L-Phe, and the enzyme also exhibits positive cooperativity for its substrate. The activation by the substrate represents the most important mechanism for PAH regulation in liver, which is believed to be of physiological significance as a way to control Phe homeostasis in blood [31, 32]. Phosphorylation of PAH at Ser16 by cAMP-dependent protein kinase (PKA) is an additional post-transcriptional regulatory mechanism that acts synergistically with substrate activation [32]. That is, L-Phe enhances the rate of phosphorylation by PKA, which in turn facilitates the activation of the enzyme by its substrate [31, 32]. Furthermore, the enzyme is also regulated by BH_4_ in a complex way. In addition to its co-substrate role, the cofactor BH_4_ also functions as a negative effector that competes with L-Phe activation, reduces the rate of phosphorylation at Ser16, and forms a stabilizing dead-end PAH-BH_4_ complex at low concentrations of L-Phe, also *in vivo* [31, 33]. Though many tetrahydropterins may substitute BH_4_ in the hydroxylation reaction, the cofactor with the natural configuration at the dihydroxypropyl side chain at C6, i.e. 1´,2´-dihydroxypropyl-5,6,7,8-tetrahydrobiopterin (6*R*-BH_4_), is the most effective at forming the stable PAH-BH_4_ complex that locks and stabilizes PAH through a large conformational change involving the N-terminal autoregulatory sequence (residues 1-33) [22, 34]. In so doing, BH_4_ acts as a *natural chaperone ligand* of PAH (see below). 

### Phenylketonuria As a Misfolding Disease

There are over 500 different PAH alleles recorded in PAHdb, with the majority corresponding to point missense mutations (63%) and small deletions (13%) [5]. During the last two decades increased evidence has been accumulated that most pathogenic mutations in *PAH* are associated with misfolding, and PKU is often considered to be a model paradigm for loss-of-function genetic metabolic diseases [35]. It is nevertheless important to mention that the HPA condition is multifactorial and PKU is a complex trait genetic disease, in which genetic and non-genetic modifiers contribute to inconsistencies in the genotype–phenotype relationships [5]. Nonetheless, in the case of PKU, there is a good correlation between the misfolding effect and the biochemical and physiological phenotypes [28, 36, 37]. Though fibril formation is clearly observed *in vitro* [38], amyloid or other fibrillar depositions which are deleterious in other protein folding defects have not been reported for PKU, and it seems that the misfolded mutants are effectively degraded by the cellular quality control system. Recent evidence from the ENU1/2 heteroallelic mouse model for HPA has shown that mutant PAH is highly ubiquitinated *in vivo* [39], supporting its targeting to proteasome-mediated degradation and/or selective autophagy [40]. The disease picture is thus mainly the outcome of lack of specific enzymatic activity, without secondary consequences of protein depositions. This further supports the choice of therapeutic strategies aiming to rescue the destabilized PAH mutants. 

During the last years, major advances have been headed by different groups, often working on multidisciplinary and multisite consortia, to investigate alternative therapeutic approaches for PKU, such as gene therapy with the functional recombinant *PAH* gene being targeted into liver or skeletal muscle [41, 42], supplementation treatment with large neutral amino acids (LNAAs) [43], enzyme replacement therapy with phenylalanine ammonia lyase (PAL), for which both injectable and oral formulations are being tested [44, 45] and the therapeutic supplementation with the PAH cofactor, BH_4_ [46] (for review see [47]). A particular promising approach aiming to correct altered splicing patterns is antisense oligonucleotide therapy [48, 49]. The large number of misfolding mutations makes PKU a disease with potential to respond to pharmacological chaperones, and this strategy has also been investigated [50, 51]. 

### Molecular, Chemical Chaperones and Pharmacological Chaperones

The term *molecular chaperones* is reserved for the large molecular machines which in an energy-dependent manner ensure the correct folding of intracellular proteins [52, 53]. Together with the ubiquitin-proteasome and the selective autophagy systems, molecular chaperones constitute the quality control system acting at different subcellular localizations, working in concert to reduce the accumulation of misfolded proteins by either refolding or destroying them [40]. There have been some recent studies which have revealed that the activity of molecular chaperones may be modulated as a selective therapeutic strategy for the treatment of misfolding diseases, notably neurodegenerative diseases (for review see [54, 55]). 

*Chemical chaperones* is a term usually associated to low molecular weight compounds such as glycerol, trehalose, glycine betaine and trimethylamine N-oxide, which can stabilize and aid to correct misfolding of proteins in general by binding to nonspecific sites [56, 57]. These compounds are osmolytes that accelerate folding and aid to bypass kinetic traps in folding pathways. Glycerol and other polyol compounds have actually been shown to increase the yield and activity of PAH mutants produced *in vitro* in prokaryote expression systems [38, 58, 59]. Other osmolytes such as 4-phenylbutyric acid (PBA) have shown therapeutic potential in a number of misfolding disorders, and PBA has been approved by the Food and Drug Administration (FDA) for the management of urea cycle disorders [60]. To our knowledge, however, the effect of chemical chaperones increasing steady-state PAH levels *in vivo* has not been reported yet. One of the limitations of using chemical chaperones for therapeutic correction of conformational diseases is its lack of specificity and the requirement of high concentrations (≥ mM), which are normally toxic for functionality *in vivo*. On the contrary, pharmacological chaperones, which are designed to specifically bind and stabilize their target protein, may be effective at low concentrations.

*Pharmacological chaperones* usually resemble natural ligands of the target proteins, and can rescue the misfolded conformers of these proteins by stimulating their renaturation or scaffolding the final folded structure [61-63]. In fact, inhibitors have been found to be effective chaperones in a number of cases [64]. In addition, compounds found through high-throughput screening (HTS) can be effective and, in the case of PKU, both HTS and derivatization of the BH_4_ scaffold have been utilized to develop stabilizing compounds with therapeutic potential [50, 51] Fig. (**3A, B**). In the case of PKU, the natural cofactor BH_4 _is itself a natural chaperone ligand that can be considered a pharmacological chaperone when given as therapeutic supplementation. 

## 2. BH_4_ AS NATURAL PHARMACOLOGICAL CHAPERONE FOR PKU

In 1999, Kure *et al.* described four patients with PAH deficiency that responded to BH_4_ loading with a reduction in plasma Phe levels [65]. The patients had normal urinary pteridine levels and DHPR activity, suggesting that they were not deficient in BH_4_ synthesis or recycling. Furthermore, molecular analysis actually confirmed PAH mutations in both alleles for these patients. This study thus revealed that BH_4_ therapy might be beneficial also to certain patients with specific PAH mutations, and this novel subtype of PAH deficiency was named BH_4_-responsive HPA/PKU [65]. Posterior studies have strengthened this notion [66, 67], but the responsive phenotype cannot be predicted solely from the genotype. Nevertheless, mild PKU mutations, with substantial residual activity, are more likely to respond to BH_4 _[67]. Responsiveness is confirmed through BH_4 _loading tests, for which conditions vary in the literature and among different clinics. Customary tests include single administrations with 20 mg BH_4_/kg body weight/day, which should cause a reduction in plasma Phe of at least 30% to classify the patient as responsive, followed by trials over several weeks, where the dose is adjusted according to the response in the patient [67]. The subset of PAH mutations and genotypes with a high probability of being BH_4_ responsive are registered in the BIOPKU: International Database of Patients and Mutations causing BH_4_-responsive HPA/PKU (http://www.biopku.org/BH4DatabasesBiopku.asp).

The efficacy and safety of BH_4_ supplementation treatment using the commercial form of the natural BH_4_, i.e. Kuvan^TM^ (sapropterin dihydrochloride, BioMarin Pharmaceutical Inc, USA) has been demonstrated in clinical trials [68]. About 40% of mild PKU patients reach a stable reduction of >30% of plasma Phe levels with this treatment, increasing their dietary Phe tolerance. 

The mechanisms underlying responsiveness to BH_4_ have been studied by several groups, and a number of possible mechanisms have been put forward: i) increase of the activity by a “Michaelis-Menten” effect, with notable relevance for catalytic defective mutant enzymes with decreased affinity for BH_4_ (high *K*_m_ for BH_4_); ii) stabilization of the mutant proteins, i.e., BH_4_ could act as a pharmacological chaperone protecting the active tetramer/dimer forms from degradation by the cellular quality control system; iii) upregulation of *PAH* gene expression levels or PAH mRNA stabilization; and iv) protection from inactivation. All together, the results indicate that the response to BH_4_ supplementation may have a multifactorial basis, and except for mechanism iii), all the other effects have been demonstrated [46, 69]. Although protection from misfolding by the pharmacological chaperone effect has received much attention, it seems that the responsiveness to BH_4_ might be largely associated to the presumed suboptimal physiological concentrations of BH_4_ normally present in hepatocytes (i.e. 5 µM; vs. a *K*_m_(BH_4_) of PAH=10-35 µM) [70]. Thus, increasing intracellular cofactor concentration will have a large effect in the activity of some mutations with sufficient residual activity to benefit from the concentration increase. In fact, recent investigations indicate that this straightforward molecular mechanism (“Michaelis-Menten” effect) preferentially contributes to the responsiveness in the ENU1/2 mice model [39].

Few studies are available on the effect of BH_4 _on TH and the TPHs, and while it has been reported that BH_4_ irreversibly inactivates [71] and aggregates TH [72], there are conflicting results in the literature on the effect of the cofactor on TH. In fact, recent results indicate that when mice are treated with BH_4_ at concentrations of 100 mg BH_4_/kg/day given orally, TH protein and activity levels increase in brain. On the other hand, BH_4_ is not very effective in crossing the blood-brain barrier, and at the typical concentrations used in the therapeutic regimes for BH_4_-responsive PKU (5-20 mg BH_4_/kg/day [2]), TH protein and activity do not seem to be significantly altered [73]. Furthermore, we have also found that oral BH_4_ supplementation, at concentrations from 20 to 100 mg BH_4_/kg/day, did not affect brain TPH2 protein and TPH activity (Thöny, B. and Martinez, A., non published results). 

## 3. DEVELOPMENT OF PHARMACOLOGICAL CHAPERONES BY VIRTUAL SCREENING APPROACHES

The usual consequence of binding a ligand to a specific binding site on the native state of a protein is an increase in protein stability [74]. Proteins, and in particular enzymes, are largely stabilized by their cofactors, substrates, and inhibitors, and a customary approach to develop pharmacological chaperones towards an unstable enzyme has been to analyze and measure the stabilizing effect of the already known inhibitors [64, 75]. Optimally these inhibitors should bind reversibly and with an affinity comparable to that of the substrates [76]. This relatively straightforward approach has led to promising compounds (in different stages in preclinical and clinical testing) for some misfolding diseases such as lysosomal storage disorders [77, 78], cystic fibrosis [79], or progressive familial intrahepatic cholestasis type 2 and 3 [80, 81]. 

In the last years, in parallel with the development of the technology in the field of bioinformatics and the advances in computational chemistry, the use of different software and *in silico* approaches has revealed to be of great aid not only in seeking pharmacological chaperones but also in drug discovery in general [82]. This computational-based search of potential drugs against specific disease-associated targets can be referred to as *virtual screening*, and there are many examples of successful stories combining virtual screening with “bench” techniques [83]. One of the main advantages when using these computational tools is the possibility of handling a large amount of compounds (from commercial libraries, databases, etc.), which increases the probability of finding hits. Nevertheless, there are remaining challenges in virtual screening and the applied algorithms, such as implementation of system flexibility and genetic diversity of the target without compromising computational time and hardware cost [82, 84]. Fig. (**4**) illustrates four methodological approaches for virtual screening which are being used in drug discovery in general and for pharmacological chaperones in particular. These methods are usually combined and applied to the drug discovery process in a synergistic way.

*Molecular docking* has long been used both by academia and the pharmaceutical industry to screen large virtual databases of compounds and score their theoretical binding affinity for a target protein of known structure. The area of interaction is usually defined around the active site in the case of enzymes. We have previously used molecular docking with the program DOCK in combination with NMR to determine the enzyme-bound conformations of the amino acid substrate and BH_2_/BH_4_ in the active site of PAH and the other aromatic amino acid hydroxylases [23, 24]. The program DOCK has also been used successfully to screen large libraries in the discovery of pharmacological chaperones for rhodopsin [85]. There are several other programs to carry out molecular docking, all of them based on algorithms that evaluate the interactions and clashes between the ligand and the protein and provide a final score. The software Glide (Schrödinger LLC) is being increasingly used for virtual screening since, differently to most other docking programs which assume a rigid 3D structure for the protein, Glide performs a “grid minimization” around the compounds with highest score [86].

The concept of *derivatization of ligands/lead optimization* normally refers to the last stage of the drug discovery process in which the initial hits obtained from a screening or any other method are progressed into drug-like compounds [87]. Basically the aim of this step is to improve and tune some of the properties of the molecule, such as lipophilicity, synthetic accessibility, absorption, distribution, metabolism, toxicity, and excretion. Lead optimization has recently also been used in the early stages of the search of pharmacological chaperones for PKU by Santos-Sierra and coworkers [51]. In this study, motivated by the accumulating evidence revealing the chaperone effect as one of the molecular mechanisms in BH_4_ responsive PKU (see above), the authors used BH_4_ as the query molecule for their shape-focused virtual screening of a large database (National Cancer Institute’s chemical library (NCI)). They used the program ROCS (Rapid Overlay of Chemical Structures), which evaluates the similarity in shape between the molecules. The software assesses the volume overlap between the reference molecule and the molecules in the database, taking into consideration only the heavy atoms. In addition, a chemical functionality overlap value is added to the equation, leading to the establishment of a score that allows the computer program to rank the potential ligands. 

In the work by Santos-Sierra *et al.*, the derivatization/lead optimization of BH_4_, complemented with molecular docking using the crystal structure of PAH and experimental surface plasmon resonance (SPR) analysis (see below), lead to two new molecules as candidates for drug development [51] Fig. (**3B**). This work thus represents a proof of principle of the strength of virtual screening in the discovery of alternative ligands with chaperoning activity for the treatment of PKU.

Another strategy within virtual screening is the *development of a pharmacophore model* which, according to IUPAC, is “an ensemble of steric and electronic features that are necessary to ensure the optimal supramolecular interactions with a specific biological target and to trigger or block its biological response” [88]. Depending on the background information available for the system of study the model can be Fig. (**4**), i) *ligand-based*, where common chemical features, representative of important interactions between the ligands and the protein target, are extracted from the structures of a set of ligands to prepare the pharmacophore model; this procedure can be applied to drug discovery even in the absence of a 3D structure for the target protein, or ii) *receptor-based*, where an analysis of the complementary chemical and spatial features of the binding site(s) – directly on the 3D structure of the target – reveals putative ligands, estimates their affinities/activities and provides the features of the pharmacophore [84]. Both approaches have given satisfactory results in a large number of virtual screenings in which the pharmacophore model is used to filter large libraries and select those with the selected features (for review see [89]). 

Virtual screening strategies [85] or the combination of virtual and limited experimental screening for pharmacological chaperones [51] may be biased towards the selection of compounds that bind into the active site, except if other sites are knowingly pre-selected in the docking or the pharmacophore modeling. On the other hand, large scale experimental HTS approaches represent a way to randomly test thousands of compounds in the shortest possible time without the *a priori* selection of ligands binding at the active site, usually as competitive inhibitors. Thus, HTS might be advantageous to discover compounds binding in various regions of the protein, with the potential for enhancing stabilization without affecting the activity [76] and for being more effective in the correction of specific mutations (*patient tailored pharmacological chaperones*). 

## 4. PHARMACOLOGICAL CHAPERONES DISCOVERED BY RANDOM SCREENING

### High-Throughput Screening

High-throughput screening (HTS) is a relatively new methodological approach in drug discovery. Using robotics, liquid handling, sensitive detectors, and computer-aided analysis, thousands and even millions of compounds can be tested for pharmaceutical activity relatively quickly (for recent reviews, see [90] and [91]). The result of a HTS provides the hits which are validated and further developed to lead compounds [92]. Most searches for pharmacological chaperones have focused on lysosomal storage diseases. For Gaucher disease, there have been several attempts to find pharmacological chaperones by HTS, but almost all the hits from initial studies based on enzyme assays employing wild-type glucocerebrosidase and fluorogenic substrates [93] failed in the hit-to-lead stage [94]. An improved HTS using a mutant enzyme extracted from the spleen of a patient has later provided more promising lead compounds [94]. Potential pharmacological chaperones have also been found by activity-based HTS for Tay-Sachs and Sandhoff diseases [75]. However, a challenge with screening based on enzyme activity is that potential pharmacological chaperones will not necessarily increase (or inhibit) the activity at standard conditions, and many of the discovered compounds with this potential are in fact very mild inhibitors [75, 95]. Moreover, in the case of PAH accessible enzyme activity assays have medium-low sensitivity and are not so suitable for up-scaling to HTS. 

**Fig. (5):**
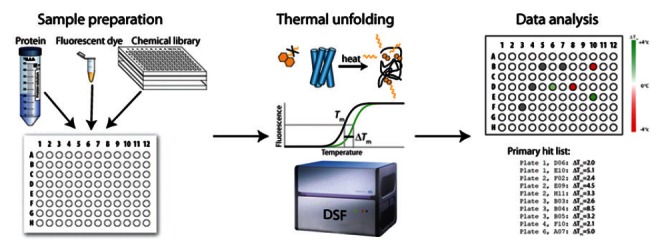
The protocol for high-throughput screening through differential scanning fluorimetry. The enzyme is mixed with the compounds and a fluorescent dye. The temperature of the samples is gradually increased and fluorescence is measured. The resulting thermal unfolding curves are analyzed and hits are picked based on their ability to stabilize the enzyme thermally. DSF, differential scanning fluorimetry.

To circumvent the problems with a high-throughput enzyme activity assay for PAH, Pey *et al.* [50] used differential scanning fluorimetry (DSF, also called thermal shift or Thermofluor^®^ stability assay) [96-98] as their primary screening for pharmacological chaperones for PAH (see Fig. **5** for an schematic description of the method). DSF is based on the fact that a ligand will stabilize the enzyme thermally [74] and lead to increased mid-point denaturation temperature (*T*_m_). The experimental setup of DSF is simple and easy to scale up. The compounds are mixed with the protein and a fluorescent probe and distributed into the wells of a microtiter. The thermal unfolding is then measured in the presence of a fluorescent probe with a conventional instrument for real-time PCR. Pey *et al.* used the fluorescent probe ANS [50, 99]; however, currently SyproOrange is more commonly used. At low temperatures, when the protein is folded, the fluorescence of this probe is quenched by water. When the temperature is increased, and the protein starts to unfold, the fluorescent probe will interact with the exposed hydrophobic patches of the protein and become unquenched. Thus, when appropriate scaling and baseline correction are applied, the fluorescence monitors the fraction of unfolded protein and the apparent *T*_m_ can then easily be obtained. All compounds which increase the *T*_m_ by more than a selected threshold value (e.g. 2 °C) are regarded as hits in the screening. As a proof of principle, Pey *et al.* screened a 1,000 compound subset of a 10,000 compound library [50]. 

As previously discussed, Santos-Sierra *et al.* applied a shape-focused *in silico* screening as the primary screening, but to verify the primary hits they used SPR [51]. SPR has become a standard in industrial and academic screening for molecules binding to proteins (see e.g. [100]). In addition, SPR has previously been used to investigate the binding and conformational changes effected by the substrate and the cofactor on PAH and TH [101-103]. Thus, SPR is a technique very suitable for experimental analysis of molecules found by *in silico* screening, and while Santos-Sierra *et al.* [51] only tested the 84 hits from the *in silico* screening, SPR could easily be used to screen a much larger library for potential pharmacological chaperones [100]. 

## 5. VERIFICATION OF HITS

After the primary screening the hits need to be validated, since some of the hits are false positives, while others are true binders but not pharmacological chaperones. After their respective screenings Pey *et al.* [50] and Santos-Sierra *et al.* [51] were left with almost the same number of potential pharmacological chaperone hits, 14 and 13, respectively. To reduce these numbers further, both groups used validation methods to exclude false positives and for secondary verification of binding. Pey *et al.* also characterized the enzyme kinetic parameters for wild-type PAH in the presence of their four principal hits Fig. (**3A**) and found that the compounds were weak inhibitors. The recent determination of the X-ray crystal structure of PAH complexed with compound IV has revealed that the compound binds to the active site iron [104], providing a molecular mechanism for the inhibitory and stabilizing effect.

An important step in the verification workflow is to analyze the effect of the compounds on the susceptibility of the protein to endogenous degradation in cells. In the case of the studies on pharmacological chaperones for PAH, it was found that the amount of immunoreactive PAH protein and PAH activity in eukaryote cells transiently expressing wild-type and mutant PAH increased at defined incubation times in the presence of the selected compounds [50, 51]. 

The efficacy of a drug cannot however be uniquely estimated from cell experiments, as it also depends on processes such as absorption, metabolism, distribution and excretion of the drug, and tests on animal models, customary mice, are necessary. Pey *et al.* tested compounds III and IV in normal mice, and administered them orally at 0.25 and 5 mg/kg/day over a period of 10 days. After the treatment the mice were sacrificed, finding an increase of immunoreactive PAH and activity in liver extracts. These studies also proved that the effect was directed at stabilizing the protein as gene expression was unchanged [50]. Santos-Sierra *et al.* treated the mild-HPA mouse model ENU1 with 6 different compounds Fig. (**3B**) given single or triple dose of 10 mg/kg/day, and used ^13^C breath test [105] in addition to measurements of blood Phe. Two of the compounds showed an increase in PAH activity relative to the reference, and one of them was twice as efficient as treatment with BH_4_ [51]. 

A final step in the validation of the hit compounds should aim to verify their specific interaction with the selected target. In the case of PAH, the possible interaction of the hits with the other enzymes of the amino acid hydroxylase enzyme family should be investigated. Calvo *et al.* tested the effect of treatment with compounds II, III and IV Fig. (**3A**), given at 5 mg/kg/day over a period of 10 days, on the brain enzymes TH and TPH2 [106]. While compound II had no effect on TH or TPH activities at the selected conditions, a significant (almost 2-fold) increase in TH activity in mouse brain extracts was induced by treatment with compound III. However, there were no measurable effects on the levels of monoamine neurotransmitter metabolites dopamine, dihydroxyphenylacetic acid, homovanillic acid, serotonin and 5-hydroxyindolacetic acid, which might reflect the strict regulation of TH *in vivo* [12, 107]. On the other hand, compound IV led to a 10–30% decrease of both brain TPH activity and monoamine neurotransmitter metabolites. Further testing of compound IV as a putative pharmacological chaperone for stabilization of PAH thus seems to require a thorough investigation of its inhibitory effect and consequent derivatization of its structure to hinder unspecific interactions.

## 6. PHARMACOLOGICAL CHAPERONES IN THE CONTEXT OF PERSONALIZED THERAPY

Along this review we have outlined the importance and promising future perspectives of pharmacological chaperones in the treatment of PKU in particular and other misfolding diseases in a more general way. In Fig. (**6**) we show a summarizing visionary workflow for patient-tailored selection of therapies (current and future), depending on the encountered PKU mutations and probability of response to pharmacological chaperones. Despite the relevance of the potential chaperones as possible drug compounds, they are only applicable in the case of mutations that lead to a misfolding state of the protein (see above). In the case of PKU such mutations are however the most prevalent ones [28].

In any case, alternative treatments for different types of mutations are needed. The combination of Guthrie’s neonatal test [7] and mutation screening allows for the earliest possible initiation of treatment avoiding the negative mental impact, and contributes to further develop the genotype-phenotype correlations in HPA/PKU. Thus, increasing understanding of specific molecular mechanisms at the individual PKU patient level lays the groundwork for effective and tailored treatment according to the genotype through a translational personalized approach, including the use of *patient tailored pharmacological chaperones*. Still, the most widely employed therapy is the institution of a low Phe diet. The advantage of this therapy is that it can be used towards all mutations with an acceptable outcome [9], as it is adjusted for each patient by monitoring Phe levels. Despite the recent improvements of the diet (e.g. a larger selection of low-Phe products with better palatability), it still presents the social burden and inconvenience of a lifelong diet and the requirement of a strict follow up with periodic controls [108], encouraging the search of therapeutic alternatives. At present, the use of BH_4_ supplementation represents a real therapeutic option for responsive patients. 

## 7. OUTLOOK

In modern medicine developing generic therapeutic approaches which in addition can accommodate the development of individualized and patient-tailored medication appears as one of the major challenges. This is even more relevant in the case of orphan or rare genetic diseases, which are the subject of little specific research. Discovery and development of pharmacological chaperones based on virtual and experimental HTS methodology, for intervention of rare misfolding diseases, such as HPA/PKU, fall within this strategy. Recent results have shown that patients harboring a subset of mild PAH mutations show normalization of blood Phe levels upon oral administration of the cofactor BH_4_. Moreover, other compounds found through HTS of diversity libraries and through computational screening using the BH_4_ scaffold also show chaperoning potential, notably for milder forms of the disease. These promising results encourage further search for more effective compounds that can rescue severe mutant forms of PAH. 

The screening, verification, and validation of hit compounds with pharmacological chaperone potential represent a challenging and arduous activity. A successful drug discovery for targeting misfolded PAH would require the integration of multidisciplinary methodological procedures spanning from chemical biology and structural/functional/thermodynamic characterization of the enzyme-compound complexes to *in vivo* studies in animal models, leading towards the initiation of clinical trials.

## Figures and Tables

**Fig. (1):**
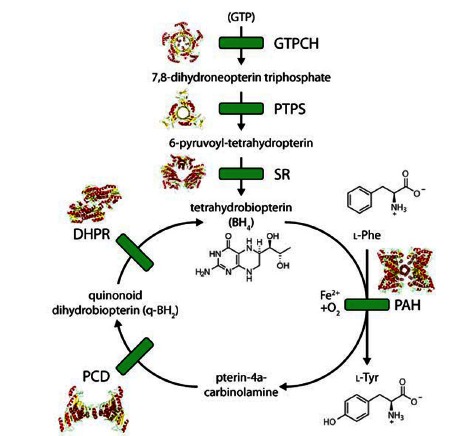
The biosynthetic and regeneration pathways of BH_4_ and the reaction catalyzed by PAH. See main text for full names of the enzymes. The 3D structures of mammalian enzyme forms are also shown (for some only truncated conformations are available).

**Fig. (2):**
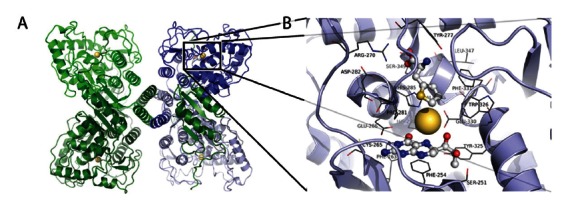
The structure of PAH. (**A**) The modeled structure of full-length tetrameric PAH (composite model prepared by combining the structures of tetrameric human PAH (residues 118-452; PDB 2PAH) and dimeric rat PAH (residues 19-427; PDB 2PHM)). (**B**) Detailed structure of PAH including thienylalanine and BH_4_ (PDB 1KW0).

**Fig. (3):**
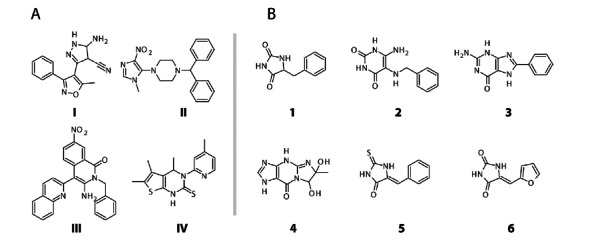
Compounds with potential pharmacological chaperone ability for PKU. (**A**) The hits from Pey *et al*. [50], where compounds **III** (3- amino-2-benzyl-7-nitro-4-(2-quinolyl)-1,2-dihydroisoquinolin-1-one) and **IV** (5,6-dimethyl-3-(4-methyl-2-pyridinyl)-2-thioxo-2,3- dihydrothieno[2,3- d]pyrimidin-4(1H)-one) showed the best potential. (**B**) The hits from Santos-Sierra *et al.*, [51], where compounds 1 (benzylhydantoin) and 2 (6-amino-5-(benzylamino)-uracil) showed the best potential.

**Fig. (4):**
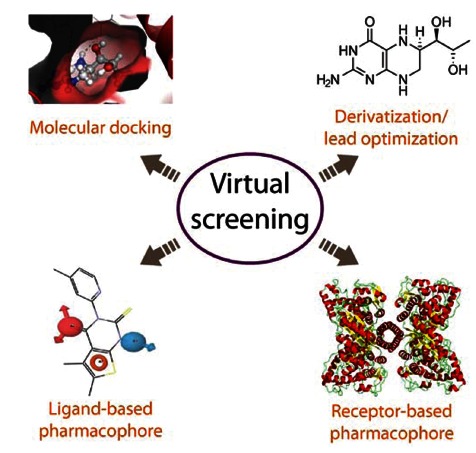
Four alternative methodological approaches for virtual screening; these methods can be combined in a synergistic way. Partly based on [84].

**Fig. (6):**
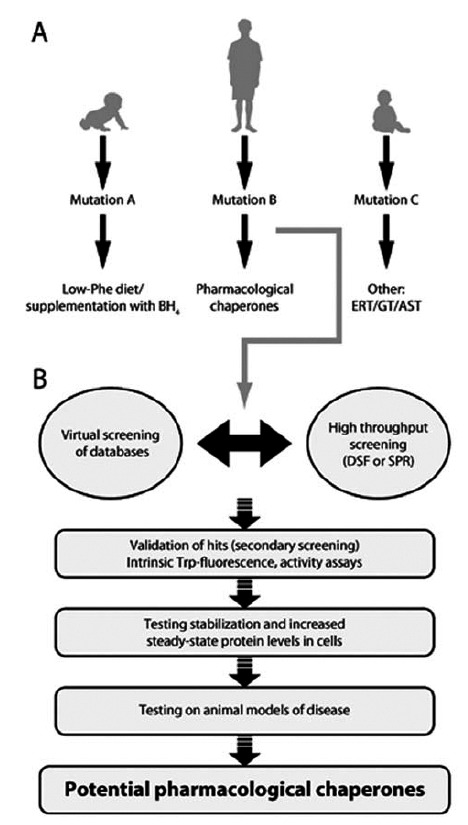
A vision for patient-tailored therapy in HPA/PKU with focus on pharmacological chaperones. **A**) The concept of genotypedependent therapy including some present (and future) alternatives in the treatment of HPA/PKU. ERT, enzyme replacement therapy; GT, gene therapy; AST, antisense therapy. **B**) Flow chart of translational approach in the process of discovery and validation of pharmacological chaperones. DSF, differential scanning fluorimetry; SPR, surface plasmon resonance.

## ABBREVIATIONS

BH_4_: = (6*R*)-L-*erythro*-5,6,7,8-tetrahydrobiopterin
HPA: = Hyperphenylalaninemia
HTS: = High-Throughput Screening
PAH: = Phenylalanine Hydroxylase
PKU: = Phenylketonuria
SPR: = Surface Plasmon Resonance
*T*_m_: = Midpoint Denaturation Temperature
